# Addressing cancer survivors’ information needs and satisfaction: a systematic review of potential intervention components for survivors with a rare cancer type

**DOI:** 10.1186/s13023-024-03403-7

**Published:** 2024-10-18

**Authors:** Tamsin Farrugia, Saskia F. A. Duijts, Carlene Wilson, Laura Hemming, Christine Cockburn, Evelien Spelten

**Affiliations:** 1 Violet Vines Marshman Centre for Rural Health Research, Rural Health School, La Trobe University, Bendigo, VIC 3552 Australia; 2 Rare Cancers Australia, 122/302-306 Bong Bong St, Bowral, NSW 2576 Australia; 3 Department of Research and Development, Netherlands Comprehensive Cancer Organisation (Integraal Kankercentrum Nederland, IKNL), Utrecht, The Netherlands; 4 Department of Medical Psychology, Amsterdam University Medical Centres, Location Vrije Universiteit, Amsterdam, The Netherlands; 5 Cancer Center Amsterdam, Cancer Treatment and Quality of Life, Amsterdam, The Netherlands; 6 Melbourne School of Population and Global Health, Melbourne University, Melbourne, VIC Australia

**Keywords:** Rare cancer, Information needs, Satisfaction with information, Supportive care, Oncology

## Abstract

**Objective:**

Providing current, evidence-based information to cancer survivors is critical for informed decision making. People diagnosed with a rare cancer report higher unmet information needs compared to common cancer survivors. However, interventions providing informational support for rare cancers are limited. Therefore, the aims of this systematic review were to identify and synthesise interventions decreasing survivors’ information needs and/or improving satisfaction with information, and to explore potential components to be included in an intervention for rare cancer survivors.

**Methods:**

Searches were conducted in PubMed, CINAHL, Embase, PsycINFO and the Cochrane Library. Studies reporting an intervention targeting information needs and/or patient satisfaction with information in survivors of any cancer type were included. Data were extracted, a quality assessment performed and findings were synthesised.

**Results:**

A total of 7012 studies were identified and 34 were included in the review. Five studies targeted patients with a rare cancer type; the remaining studies included common cancer survivors. Interventions varied in relation to the mode of information provision, timing of intervention delivery, and the intervention provider. The most promising interventions included face-to-face communication and written material and were delivered by a nurse. All rare cancer studies were designed around a web-based program, but none of them improved outcomes.

**Conclusions:**

Interventions targeting information needs and/or patient satisfaction with information in rare cancer survivors are lacking. Future studies should focus on this underserved group, and successful aspects of interventions for common cancer survivors should be considered for inclusion when designing an intervention for rare cancer survivors.

**Supplementary Information:**

The online version contains supplementary material available at 10.1186/s13023-024-03403-7.

## Background

A cancer diagnosis presents a life-altering event, triggering a cascade of emotional, physical, social and financial challenges. In the face of such an event, the provision of relevant, tailored and evidence-based information can influence a cancer patient’s ability to understand their diagnosis and navigate their trajectory by supporting them to make informed decisions about their treatment and care [1].

Rare cancers, defined as those with an incidence fewer than 6 diagnoses per 100,000 people per year, account for an estimated 13% of new cancers diagnosed and 14% of all cancer deaths in Australia [2]. Patients diagnosed with a rare cancer face unique challenges on top of those already experienced by those with a common cancer. These challenges may include receiving an incorrect and/or delayed diagnosis, being confronted with limited availability of evidence to guide decision-making and treatment options, difficulty in finding expert clinical knowledge, and having poorer access to clinical trials [3, 4]. As a result, rare cancers are often diagnosed at a more advanced stage than common cancers, presenting much poorer prognosis and lower quality of life [5]. Further, rare cancer survivors report greater unmet information needs compared to common cancer survivors [6], desiring specific information to guide the intricacies of their diagnosis.

The vast heterogeneity of rare cancers proves an immense challenge in providing relevant and tailored information unique to the individual. Further, Yatim, et al. [7] reported on the importance of communicating information to ensure that patient understanding and satisfaction is achieved. Previous studies have shown an association between dissatisfaction with information and anxiety and depression [8], emphasising the importance of patient satisfaction with information provided when meeting information needs of (common and rare) cancer survivors.

There are many sources of information available to cancer survivors. Patients report a preference for retrieving information from multiple sources to gain knowledge about their disease [1, 9, 10]. Also, they have a preference to be informed by their primary physician [1, 11]. Yet recently, the internet has become an important source of information for cancer survivors, given the accessibility, ease of using and anonymity of searching online [9, 10]. Patients need to be aware of the large amount of misinformation available online and navigate this to ensure they seek relevant information to their situation [12].

To meet the unmet information needs of rare cancer survivors, developing targeted interventions is important. However, previous research has predominantly focused on identifying and addressing the unmet information needs amongst people diagnosed with a common cancer. Unfortunately, there is limited evidence describing interventions that reduce unmet information needs among rare cancer survivors, thereby posing a critical gap in rare cancer supportive care. It is essential that healthcare professionals understand the information needs of rare cancer survivors including how to appropriately deliver tailored information in order to ensure unmet information needs, and satisfaction with information, are met.

The aims of the current systematic review were to identify and synthesise interventions improving cancer survivors’ information needs and/or satisfaction with information, and to explore potential components to be included in an intervention for rare cancer survivors.

## Methods

### Search strategy and eligibility criteria

A systematic search was conducted in the databases PubMed, CINAHL, Embase, PsycINFO and the Cochrane Library. Studies were restricted to those published from January 2011 until February 2024, based on the establishment of the definition of rare cancers in 2011 [13]. Studies were identified using search terms based on the PubMed strategy that incorporated a combination of medical subject headings (MeSH) and free text terms relating to cancer, information needs, supportive care and interventions. The search syntax was modified for each database as appropriate (Supplementary Information Tables S1-S5).

To be included, studies had to describe an intervention that addressed information need(s) among adult (≥ 18 years) cancer survivors, regardless of tumour type or stage of disease, and reported on one or more of the outcomes, i.e. unmet information needs and/or patient satisfaction with information provided. Studies using both quantitative and qualitative methodologies were eligible for inclusion. Studies were restricted to English language and full text availability. Studies were excluded from the review if: 1. the study population included adults without a cancer diagnosis (i.e. carers); 2. the intervention focussed on supportive care in general, without analysing information needs separately, or provided information about one specific issue only (e.g. dietary advice or physical activity) to ensure the review focussed on general information needs; 3. the study design was a systematic literature review, non-systematic review (e.g. scoping or narrative), conference proceeding (e.g. abstracts, posters), editorial or case report; and 4. other reasons for ineligibility were present (e.g. no full text available).

### Selection method

Articles identified from all databases were downloaded into a reference management software program (Endnote) and duplicates were removed. Next, articles were exported into ASReview, a machine learning tool for systematic reviews that supports the title/abstract screening process [14]. In ASReview, the user prescribes the inclusion and exclusion criteria of a specified research question and the tool assesses each article for relevance, presenting the most relevant articles first for authors’ consideration. Previous research has explored the effectiveness of various stopping criteria approaches utilised by automated machine learning applications, including ASReview [15]. One such method is heuristic stopping criteria, at which the reviewer ceases reviewing on ASReview once a specified number of consecutive irrelevant articles has been seen, assuming that the probability of a relevant article from the remaining unreviewed articles is low [16]. Based on heuristic stopping criteria, as well as discussion among authors, it was agreed that abstract screening would cease after 70 consecutive irrelevant articles were identified.

Abstracts exported to ASReview were initially screened by one author (TF). Relevant abstracts were imported to Covidence for screening by a second reviewer (SD, CW, CC or ES) to ensure rigour. The included studies were independently screened in full for eligibility by two authors (TF and either SD, CW, CC or ES). Where required, the relevance of an article was discussed between two reviewers for agreement. The remaining studies were included for further analysis. Reference lists of included studies were reviewed to identify additional relevant studies.

### Data extraction

Extraction was undertaken independently in Covidence by two authors (TF and either SD, CW, CC or ES). The following data were extracted: study characteristics (including authors, title and year of publication, country, study design), participant characteristics (including type of cancer diagnosis), and study outcomes (including measures used to assess outcomes). Intervention characteristics (including intervention components used and intervention provider) were also extracted, where intervention components were defined as the mode of information delivery, i.e. written material, in person, verbally via telephone follow-up, and/or information provided through multi-media sources or from a web-based program. Further, the intervention provider was defined as the individual/s delivering the intervention, including oncology nurse, other health professional, or the intervention relied on the patient to self-navigate the intervention (i.e. they were patient-led). Interventions may have included multiple intervention providers, depending on the components used.

### Quality assessment

The quality of included studies was assessed independently by two authors (TF and either SD, CW, CC or ES) using the Cochrane Risk of Bias tool to assess randomised controlled trials (RCTs) [17], the Critical Appraisal Skills Programme checklist for qualitative studies [18], and the Joanna Briggs Institute checklists for cohort studies, cross-sectional and quasi-experimental (non-randomised) studies [19]. All studies were included in this review, regardless of their quality, due to the importance of analysing all data available on this topic.

## Results

**Fig. 1:**
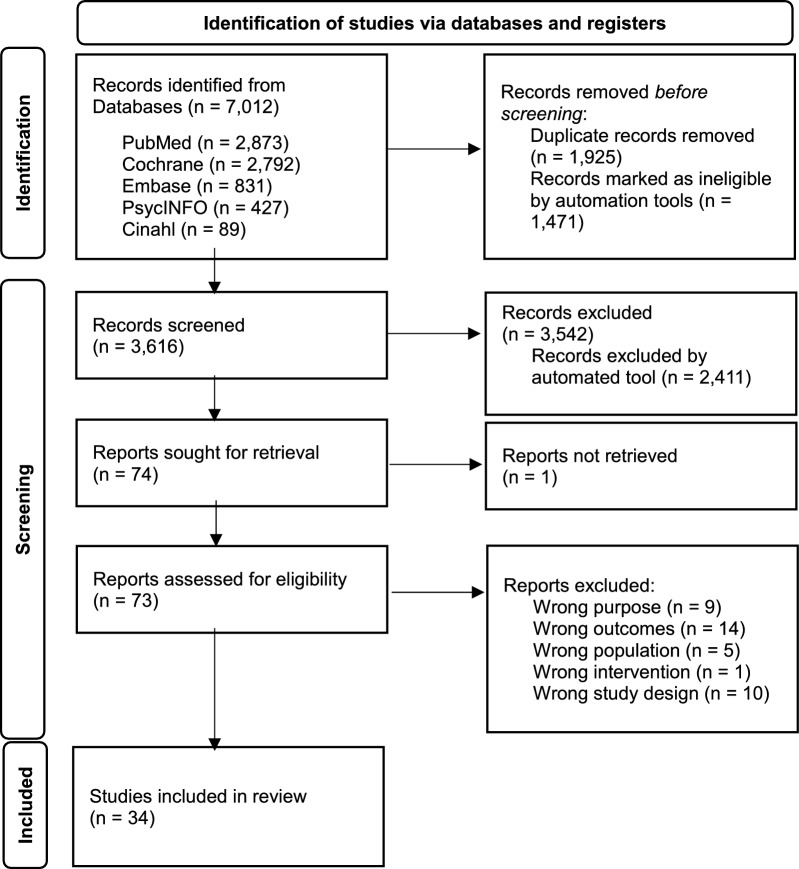
PRIMSA diagram

The search retrieved a total of 7,012 studies. After the removal of duplicates, a total of 3,616 articles remained for title and abstract screening. The threshold of 70 irrelevant abstracts was reached after screening 1,131 abstracts. Of these, 73 potentially eligible articles were reviewed in full, of which 34 met the inclusion criteria and were included in the review (Fig. 1) [20–53].

### Study characteristics

Studies were conducted in Europe (n = 14), North America (n = 10), Australia (n = 8) and Asia (n = 2). Twelve studies were RCTs [21, 22, 24, 25, 31, 36, 37, 39–41, 46, 51], eight were cohort studies [20, 30, 32, 33, 42–44, 49], five adopted a cross-sectional design [26, 27, 45, 50, 53], three were qualitative [29, 38, 52], five were quasi-experimental (non-randomised) studies [23, 28, 34, 35, 48] and one study adopted a mixed-method design [47]. More than half of the studies included were assessed as either moderate quality [20, 23, 27, 30, 37, 38, 42, 43, 46, 49] or high quality [25, 28, 29, 34, 36, 40, 48, 52]. Studies assessed as having low methodological quality [21, 22, 24, 26, 31–33, 35, 39, 41, 44, 45, 47, 50, 51, 53] were found to have high risk of bias. The study characteristics of included studies are outlined in Table 1.

**Table 1:** Study characteristics of included studies

Author, Year	Country	Study design	N	Age (years)	Sex (female %)	Diagnosis	Duration/follow-up	Outcome (measure)	Quality rating
*Studies conducted among people diagnosed with a common cancer*									
Bender, et al. [20]	Canada	Cohort	51	M 65.2 (SD 7.1)	14.7%	Prostate	3 months	SWI (PSN-I and 13-item satisfaction scale)	Moderate
Chee, et al. [22]	USA	RCT	99 (IG: 50; CG: 49)	M 52.5 (SD 12.5)	100%	Breast	3 months	UIN (SCNS-SF34)	Low
D’Souza, et al. [23]	Canada	Non-randomised	103 (IG: 50; CG: 53)	IG: M 57.9 (SD 11.1) CG: M 61.7 (SD 14.6)	IG: 30% CG: 20.8%	Head and neck	6 months	SWI (SCIP)	Moderate
Dieng, et al. [25]	Australia	RCT	24 (IG: 12; CG: 12)	IG: M 56.7 (SD 14) CG: M 61 (SD 10.5)	IG: 33% CG: 50%	Melanoma	1–6 months	SWI (Scale from 0–10)	High
Fang, et al. [27]	USA	Cross-sectional	55	M 61.1 (SD 9.8)	24.5%	Head and neck	2 weeks	SWI (5-point likert scale)	Moderate
Fox, et al. [28]	Australia	Non-randomised	14	40% 50–64	47%	Melanoma	6 months	UIN (SCNS-SF34); SWI (5-point Likert scale)	High
Hauffman, et al. [29]	Sweden	Qualitative	15	M 59 (range: 37–69)	70%	Combination (breast, prostate, colorectal)	24 months	SWI (Semi-structured interviews)	High
Heisig, et al. [30]	Germany	Cohort	137	M 56.0 (SD 10.6)	100%	Breast	3 months	SWI (SIMSC)	Moderate
Jefford, et al. [31]	Australia	RCT	221 (IG: 110; CG: 111)	IG: M 62.1 (SD 11.4) CG: M 63.1 (SD 12.0)	IG: 47.7% CG: 49.1%	Colorectal	6 months	UIN (CaSUN); SWI (5-point Likert scale survey)	Low
Jefford, et al. [32]	Australia	Cohort	10	M 55 (range: 35–71)	50%	Colorectal	Up to 7 weeks	UIN (CaSUN); SWI (Semi-structured interviews)	Low
Kasparian, et al. [33]	Australia	Cohort	19 (10 HR, 9 MR, 10 HP)	HR, MR: M 58.1 (SD 10.6) HP: M 42.8 (SD 9.4)	HR: 50% MR: 67% HP: 90%	Melanoma	NA	UIN (CaSUN); SWI (One Y/N question)	Low
Kim, et al. [34]	Canada	Non-randomised	100	Phase 1: 40% 61–70 Phase 2: 41% 61–70	Phase 1: 19.5% Phase 2: 24.4%	Head and neck	1 year	SWI (7 questions, 5-point Likert scale and 3 open-ended questions)	High
Kotronoulas, et al. [35]	Scotland	Non-randomised	10	M 51.8 (SD 14.2)	80%	Melanoma	4 months	UIN (SCNS-SF34-melanoma)	Low
Li, et al. [36]	China	RCT	140 (IG: 70; CG: 70)	Median 62 (range: 22–89)	IG: 22.8% CG: 28.5%	Head and neck	6 months	UIN (CNAT)	High
Malmström, et al. [37]	Sweden	RCT	82 (IG: 41; CG: 41)	IG: M 64.4 (SD 9.4) CG: M 68.5 (SD 9.4)	IG: 26.8% CG: 17.1%	Oesophageal	6 months	UIN and SWI (EORTC-QLQ-INFO25)	Moderate
Martin, et al. [39]	USA	RCT	140 (IG: 73; CG: 67)	M 53 (SD 10.5)	65.7%	Combination (breast, colorectal, hematologic, lung, head and neck, 21.9% other)	6 months	UIN (18 questions, 4-point Likert scale)	Low
Martin, et al. [38]	England	Qualitative	6	M 35 (range: 29–45)	0%	Testicular	6 weeks	UIN (Semi-structured interview)	Moderate
O’Connor, et al. [40]	Northern Ireland	RCT	76 (IG: 43; CG: 33)	IG: M 63.1 (SD 10.7) CG: M 68.3 (SD 9.3)	35.5%	Colorectal	6 months	SWI (PSCaTE)	High
Perfors, et al. [41]	Netherlands	RCT	154 (IG: 77; CG: 77)	IG: M 61.8 (SD 11.4) CG: M 59.3 (SD 12.2)	IG: 74% CG: 75.3%	Combination (breast, colorectal, gynaecological, lung, melanoma)	3 months	SWI (EORTC INPATSAT-32 and NRS)	Low
Samoil, et al. [42]	Canada	Cohort	248 (126 patients; 122 caregivers)	P: M 59.5 (SD 10.1) C: M 50.9 (SD 17)	P: 100% C: 48%	Ovarian and vaginal	NA	SWI (One Y/N question)	Moderate
Serena, et al. [43]	Switzerland	Cohort	46	M 60.3 (SD 8.9)	65%	Lung	Up to 80 days	UIN (SCSST)	Moderate
Singleton, et al. [44]	Australia	Cohort	841	M 58.8 (SD 9.8)	99.6%	Breast	3 months	SWI (5-point Likert scale)	Low
Skrutowski, et al. [45]	Canada	Cross-sectional	34	M 54.8	100%	Breast	2 months	SWI (Evaluation form (14 questions)	Low
Tran, et al. [47]	Canada	Mixed methods	8	Range: 48–89	75%	NA	3 months	SWI (Questionnaire 5-point Likert scale and interview)	Low
van der Meulen, et al. [48]	Netherlands	Non-randomised	48 (IG: 22; CG: 26)	IG: M 64 CG: M 69	IG: 32% CG: 42%	Head and neck	2.5 months	UIN (PINQ); SWI (SCIP)	High
Villarreal-Garz, et al. [50]	Mexico	Cross-sectional	134	M 35 (SD 4.1)	100%	Breast	NA	SWI (Questionnaire including 8 open-ended and 35 multiple choice questions)	Low
White, et al. [51]	Australia	RCT	379 (IG: 202; CG: 177)	IG: M 43.6 (SD 5) CG: M 43.9 (SD 5.3)	100%	Breast	6 months	UIN (SCNS-BC)	Low
Williamson, et al. [52]	England	Qualitative	32 (IG: 25; 7 CNS)	Median 67 (range: 52–82)	100%	Endometrial	Up to 12 months	SWI (Semi-structured interviews)	High
Yamaki, et al. [53]	Japan	Cross-sectional	447 P, 216 F	P: 52% 60–79 F: 47.7% 40–59	P: 59.3% F: 26.9%	NA	Unclear	SWI (12-item questionnaire)	Low
*Studies conducted among people diagnosed with a rare cancer*									
Bouma, et al. [21]	Netherlands	RCT	20 (IG: 10; CG: 10)	IG: Median 59.5 (41–74) CG: Median 64.0 (45–74)	IG: 60% CG: 50%	Neuroendocrine	12 weeks	SWI (EORTC-QLQ-INFO25)	Low
de Hosson, et al. [24]	Netherlands	RCT	91 (IG: 46; CG: 45)	IG: M 62 (SD 10) CG: M 63 (SD 7)	IG: 41% CG: 51%	Neuroendocrine	12 weeks	SWI (EORTC-QLQ-INFO25)	Low
Ector, et al. [26]	Netherlands	Cross-sectional	203	66.2% ≥ 55	54.8%	Chronic Myeloid Leukemia	3 years	UIN (EORTC-QLQ-INFO25); SWI (EORTC-QLQ-C30 and EORTC-QLQ-CML24)	Low
Stevenson, et al. [46]	Australia	RCT	77 (IG: 38; CG: 39)	IG: M 51 (SD 15) CG: M 49 (SD 16)	IG: 43% CG: 20%	Haematological	Up to 12 weeks	UIN (SCNS-SF34)	Moderate
Verweij, et al. [49]	Netherlands	Cohort	108 (IG: 75; CG: 33)	IG: 60.3% 18–64, 39.7% ≥ 65 CG: 36.4% 18–64, 63.6% ≥ 65	IG: 37.8% CG: 63.6%	Chronic Myeloid Leukemia	6 months	UIN (EORTC QLQ-INFO25) SWI (EORTC QLQ-CML24)	Moderate

N: Number of participants; RCT: Randomised Controlled Trial; UIN: Unmet information needs; SWI: Satisfaction with information; IG: Intervention Group; CG: Control Group; M: Mean, SD: Standard Deviation; NA: Not Available; P: Patients; F: Families; HR: High-risk patients; MR: Moderate-risk patients; HP: Health professionals; C: Caregivers, PSN-I: Patient Satisfaction with Navigator Interpersonal Relationship Scale (Satisfaction with A: amount and content of info, and B: form and timing of info); SCNS-BC: Supportive Care Needs Survey—Breast Cancer; SCNS-SF34: Supportive Care Needs Survey—Short Form; SIMSC: Satisfaction with Information about Medicines Scale Comprehension; CaSUN: Cancer Survivors' Unmet Needs; PSCaTE: Patient Satisfaction with Cancer Treatment Education; PCSQ: Preparedness for Colorectal Cancer Surgery Questionnaire; CNAT: Comprehensive Needs Assessment Tool in Cancer for Patients; SCSST: Supportive Care Needs Survey Screening Tool; SCIP: Satisfaction with Cancer Information Profile; PINQ: Patient Information Need Questionnaire; EORTC-QLQ-INFO: European Organisation for Research and Treatment of Cancer’s Quality of Life Information scale; NRS: Numeric Rating Scale

### Patient characteristics

In total, 3,893 participants were included across the 34 studies. Most studies were conducted in common cancers, including three studies that included a combination of common cancers [29, 39, 41], and three studies that included a participant cohort consisting of common cancer patients and either families [53], health professionals [33] or caregivers [42]. Five studies were performed in patients with a rare cancer type, including neuroendocrine tumours (n = 2) [21, 24], chronic myeloid leukemia (n = 2) [26, 49] and haematological cancer (n = 1) [46]. One study was performed in a less common cancer (testicular cancer) [38] and one study included participants with gynaecological cancer, including both common types and rarer forms [42]. Two studies did not specify all participants’ tumour types [47, 53].

### Intervention characteristics and outcomes

Eight studies assessed the effect of the intervention on (unmet) information needs [22, 35, 36, 38, 39, 43, 46, 51], 19 studies assessed patient satisfaction with information provided by the intervention [20, 21, 23–25, 27–30, 34, 40–42, 44, 45, 47, 50, 52, 53], and seven studies explored the intervention effect on both outcomes [26, 31–33, 37, 48, 49].

Of all studies that measured a change in information needs, in eight studies reduced unmet information needs were reported [22, 32, 33, 35–38, 43]. Of the studies that assessed patient satisfaction with information provided by the intervention, in 20 studies, high satisfaction with the intervention was shown [20, 23, 25, 27–34, 37, 40, 42, 44, 45, 47, 50, 52, 53]. Studies were implemented across all stages of the cancer pathway, with three quarters performed during the treatment and post-treatment phase [20, 22, 26–28, 30–32, 34, 36–40, 42–52]. The intervention characteristics of included studies are outlined in Tables 2 and 3.

**Table 2:** Intervention characteristics of studies assessing change in information needs

Author, Year	Study findings	Intervention component/s					Comparison	Phase in pathway			Provider
		Written	Face-to-face	Telephone follow-up	Web-based	Multi-media		Diagnosis	Treatment	Post-treatment	
*Studies conducted among people diagnosed with a common cancer*											
Kasparian, et al. [33] †	Reduced unmet information needs	Y					NA	X			Patient driven
Kotronoulas, et al. [35]	Reduced unmet information needs	Y	Y				NA	X			Nurse
Serena, et al. [43]	Reduced unmet information needs	Y	Y	Y			NA		X		Nurse
Li, et al. [36]	Reduced unmet information needs		Y	Y		Y	Usual care		X	X	Nurse; Members of the intervention team
Chee, et al. [22]	Reduced unmet information needs				Y		Access to ACS website			X	Patient driven; Bilingual interventionist
Jefford, et al. [32] †	Reduced unmet information needs	Y	Y	Y		Y	NA			X	Nurse
Malmström, et al. [37] †	Reduced unmet information needs	Y	Y	Y			Usual care			X	Nurse
Martin, et al. [38]	Reduced unmet information needs		Y			Y	NA			X	Nurse
White, et al. [51]	No change in unmet information needs	Y		Y	Y		Usual care	X	X		Patient driven
Martin, et al. [39]	No change in unmet information needs	Y	Y				NA		X	X	Nurse
Jefford, et al. [31] †	No change in unmet information needs	Y	Y	Y		Y	Usual care			X	Patient driven; Nurse
van der Meulen, et al. [48] †	No change in unmet information needs	Y	Y				NA			X	Nurse
*Studies conducted among people diagnosed with a rare cancer*											
Stevenson, et al. [46]	No change in unmet information needs			Y	Y		Usual care	X	X		Patient driven
Ector, et al. [26] †	No change in unmet information needs				Y		NA		X	X	Patient driven
Verweij, et al. [49] †	No change in unmet information needs				Y		Access to website without support		X	X	Patient driven; Project team for instructions

^†^ Studies assessing change in information needs and satisfaction with information provided

**Table 3:** Intervention characteristics of studies assessing patient satisfaction with information provided

Author, Year	Study findings	Intervention component/s					Comparison	Phase in pathway			Provider
		Written	Face-to-face	Telephone follow-up	Web-based	Multi-media		Diagnosis	Treatment	Post-treatment	
*Studies conducted among people diagnosed with a common cancer*											
D’Souza, et al. [23]	Improved patient satisfaction	Y				Y	Usual care	X			Nurse
Dieng, et al. [25]	Improved patient satisfaction	Y		Y			Usual care	X			Psychologist
Hauffman, et al. [29]	Improved patient satisfaction				Y	Y	Usual care	X			Nurse, Psychologist
Kasparian, et al. [33] †	Improved patient satisfaction	Y					NA	X			Patient driven
Skrutowski, et al. [45]	Improved patient satisfaction	Y					NA	X	X		Patient driven
Villarreal-Garz, et al. [50]	Improved patient satisfaction	Y	Y		Y	Y	NA	X	X		Program navigator
Kim, et al. [34]	Improved patient satisfaction	Y					NA		X		Nurse
Tran, et al. [47]	Improved patient satisfaction				Y		NA		X		Patient driven
Samoil, et al. [42]	Improved patient satisfaction		Y				NA		X		Nurse
Bender, et al. [20]	Improved patient satisfaction			Y	Y		NA		X	X	Peer navigators
Fang, et al. [27]	Improved patient satisfaction				Y		NA		X	X	Patient driven
Heisig, et al. [30]	Improved patient satisfaction	Y	Y				NA		X	X	Trained professionals
Fox, et al. [28]	Improved patient satisfaction			Y			NA			X	Social worker/ Counsellor
Jefford, et al. [31] †	Improved patient satisfaction	Y	Y	Y		Y	Usual care			X	Patient driven; Nurse
Jefford, et al. [32] †	Improved patient satisfaction	Y	Y	Y		Y	NA			X	Nurse
Malmström, et al. [37] †	Improved patient satisfaction	Y	Y	Y			Usual care			X	Nurse
O’Connor, et al. [40]	Improved patient satisfaction	Y	Y				Usual care			X	Nurse
Singelton, et al. [44]	Improved patient satisfaction					Y	NA			X	Unclear
Williamson, et al. [52]	Improved patient satisfaction	Y		Y			Usual care			X	Nurse
Yamaki, et al. [53]	Improved patient satisfaction	Y	Y				NA				Cancer Information Specialists
Perfors, et al. [41]	No improvement in patient satisfaction		Y				Usual care	X			GP, Nurse
van der Meulen, et al. [48] †	No improvement in patient satisfaction	Y	Y				NA			X	Nurse
*Studies conducted among people diagnosed with a rare cancer*											
Bouma, et al. [21]	No improvement in patient satisfaction		Y		Y		Usual care	X			Patient-driven. Follow-up with medical oncologist, nurse or other relevant HCP
de Hosson, et al. [24]	No improvement in patient satisfaction				Y		Usual care	X			Patient-driven; Physicians, oncology nurses, medical oncologists
Ector, et al. [26] †	No improvement in patient satisfaction				Y		NA		X	X	Patient driven
Verweij, et al. [49] †	No improvement in patient satisfaction				Y		Access to website without support			X	Patient driven; Project team for instructions

^†^ Studies assessing change in information needs and satisfaction with information provided

**Interventions addressing information needs**.

Six studies reporting a reduction in unmet information needs consisted of a face-to-face component with information being delivered by an oncology nurse [32, 35–38, 43]. In addition to an in-person component, studies also included telephone follow-up support [32, 36, 37, 43], written information [32, 35, 37, 43], or a multi-media component [32, 36, 38]. The remaining two interventions were designed around one intervention component and were patient-led.

Four of the eight studies showing an improvement in information needs were assessed as moderate to high quality, based on low risk of bias [36–38, 43]. The remaining four studies were assessed as low quality due to lack of blinding of participants and/or personnel, no justification of sample size, the intervention only being assessed once over time, lack of comparison group and incomplete follow-up [22, 32, 33, 35].

Studies showing no improvement in information needs mainly consisted of multiple modes of information delivery. The most commonly used components included written material [31, 39, 48, 51], face-to-face communication [31, 39, 48, 49] and web-based programs [26, 46, 49, 51]. Studies showing no improvement in information needs were also mainly patient-led [26, 31, 46, 49, 51]. Most of these studies were assessed as low quality [26, 31, 39, 51].

**Interventions addressing patient satisfaction with provided information**.

Twelve studies that reported an improvement in satisfaction with provided information included multiple modes of delivery [20, 23, 25, 29–32, 37, 40, 50, 52, 53], with the provision of written material and verbal communication (either in-person or telephone follow-up) being the most promising for intervention success. Interventions varied in terms of the provider. Specifically, they were delivered by a nurse in nine studies [23, 29, 31, 32, 34, 37, 40, 42, 52] and were patient-led in five studies [27, 31, 33, 45, 47].

Of the studies showing an improvement in satisfaction with provided information, twelve were assessed as high quality evidence while eight were reported as low quality due to poor allocation concealment, lack of blinding, lack of sample size justification, intervention not being assessed more than once over time and potential confounding variables not adjusted for [31–33, 44, 45, 47, 50, 53].

The six studies not reporting an improvement in satisfaction with information focused on face-to-face communication [21, 41, 48, 49] and/or a web-based program [21, 24, 26, 49]. Four of these interventions were patient-led [21, 24, 26, 49]. The majority (n = 5) of studies not showing an improvement in satisfaction were assessed as being low quality evidence due to lack of blinding, lack of sample size justification and potential confounding variables not adjusted for [21, 24, 26, 41, 49].

**Interventions performed among rare cancer survivors**.

No studies performed in rare cancer survivors were successful at reducing unmet information needs and/or improving patient satisfaction with provided information [21, 24, 26, 46, 49]. Of the five studies conducted among rare cancer survivors, three studies [21, 24, 26] were assessed as being low quality of evidence, due to lack of blinding of participants and personnel, no justification of sample size, insufficient timeframe to see association between intervention and outcome, and no statistical adjustment for potential confounding variables.

All interventions conducted amongst rare cancer survivors were patient-led and included a website to disseminate information. In addition to a website, two studies also provided verbal information through in-person follow-up with the medical oncologist or oncology nurse [21], or optional telephone follow-up support by a nurse [46]. However, no survivors contacted this optional nurse support service.

## Discussion

### Main findings

In this review, we identified a total of 34 studies exploring interventions targeted to reduce unmet information needs and/or improve satisfaction with information provided in cancer survivors. Of the studies included, 75% (25.5/34 studies) were effective. No studies performed among rare cancer survivors improved either information needs and/or improve satisfaction with information provided. The most promising interventions, regardless of outcome, consisted of in-person communication, the provision of written material and were delivered by a nurse. While interventions designed around a web-based program were successful among patients diagnosed with a common cancer, this was not the case for those diagnosed with a rare cancer. As interventions varied in relation to the number of, and type of, components used, timing of intervention delivery, and the intervention provider, this review cannot present a clear, stand-alone approach in how best to deliver information to (rare) cancer survivors. However, this review serves as a foundational step in developing tailored interventions specifically designed for rare cancer survivors and should be considered when planning future research.

### Interpretation of findings

Interventions incorporating either verbal communication and/or the provision of written material were found to have a successful effect on the outcome of interest. In particular, the inclusion of a face-to-face component was the most promising component of ensuring unmet information needs were met. In line with previous studies, cancer patients continuously report the value and preference for speaking with healthcare professionals for information access [1, 9]. In-person communication ensures patients can receive direct, clear and tailored information from their healthcare professional, and allows the space to address patient concerns in real time. Li et al. (2020) reported that in-person communication establishes a sense of trust between a cancer patient and healthcare provider, where patients desire a balance between receiving the truth and hope [54]. However, in rare cancers, limited clinical expertise may prove a challenge in ensuring patients receive accurate and tailored information from their healthcare provider, potentially affecting trust [55]. As such, face-to-face communication is particularly important for rare cancer survivors, as they are already confronted with several challenges and uncertainty during their diagnostic and treatment pathway [55].

Interventions that incorporated the provision of hard-copy, written material were more likely to improve outcomes compared to those without, particularly for those interventions reporting an improvement in satisfaction with provided information. A recent national survey [9] reported that cancer patients overwhelmingly preferred information in the form of written booklets (83%), reporting their appreciation in the flexibility to stop and start when learning new information (58%). Further, previous studies highlighted the benefit of written material as it reiterates important information from consultations that may be forgotten or remembered incorrectly [56, 57], ensuring the patient has the opportunity to review the information in their own time when convenient to them [34]. While the provision of written information may benefit rare cancer survivors, this is difficult to determine conclusively because no interventions targeting rare cancer survivors exclusively provided written information. This may reflect the paucity of written material produced about specific rare cancers.

Although in-person communication and written material were found to be the most promising components for improving information needs and/or satisfaction with information, findings in this review showed that a range of other intervention components may also be effective. That is, telephone follow-up support and multimedia components seem to have some potential at improving outcomes for the cancer patient. In line with findings from previous studies [1, 7, 9], it is not surprising that patients desire information from multiple sources as they value the availability of different formats, providing various types and amounts of information. Additionally, there is emerging evidence that highlights the popularity of the internet for cancer information [9, 10], with web-based education proving an economical and efficient way of reaching a wide audience [58]. However, findings in this review clearly indicated that web-based programs alone are effective for those diagnosed with a common cancer, yet not successful for the rare cancer patient. Ector et al. [26] stated that rare cancer patients may require specific information tailored to the individual’s age, education level and time since diagnosis, which is difficult to address through a stand-alone web-based program. Further, Drabbe et al. [3] reported that 23% of sarcoma patients desired more information across 280 specific topics, highlighting the breadth of the information needs amongst those living with a sarcoma. Thus, the heterogeneity and unique needs of rare cancer survivors should be considered when developing an intervention to reduce unmet information needs or improve satisfaction with provided information.

Finally, studies identified in this review were inconsistent in relation to the number and types of components used, delivery of the intervention and timing of intervention delivery, suggesting there is no clear approach in how best to meet cancer survivors’ unmet information needs and/or satisfaction with information. Notably, most interventions were implemented at a particular point in the cancer trajectory, however, there was limited rationale provided for the timing of delivery. Specifically for rare cancers, there is limited supportive care research undertaken in the diagnosis and treatment phase of the cancer pathway, with most studies exploring unmet information needs conducted in the post-treatment phase [6]. This is also evident in our findings, with only two of the five rare cancer studies conducted in the diagnosis phase. Additionally, a considerable number of studies were implemented by various healthcare disciplines, particularly for studies reporting an improvement in patient satisfaction with information. This makes it difficult to ascertain a clear path for improving information delivery to (rare) cancer survivors.

### Strengths and limitations

To our knowledge, this is the first systematic literature review synthesising interventions improving cancer survivors’ information needs and satisfaction with information. In particular, the consideration of findings for the purpose of developing an intervention for rare cancer survivors has not previously been reported and can be considered a strength.

Studies included in this review showed various flaws in their methodological rigour, including a lack of blinding, insufficient sample size justification, the lack of a control group and intervention(s) (components) not being assessed over time. Ultimately, the quality of 50% the studies included were assessed as low, and as such, caution should be taken when interpreting these results. In particular, three of the five rare cancer studies were assessed as low quality.

Further, no study included in the review assessed the impact of individual intervention components on outcomes measured, thereby making it difficult to ascertain whether the effect of the intervention was due to the study characteristics (i.e. study design or participants involved) or individual intervention components.

Finally, there is a difference between countries in relation to what is considered a rare cancer, due to incidence rates and total population, despite all studies applying the RARECARE definition. However, in this review, the rarity of the cancer, and intervention success, was interpreted based on the country of publication and, therefore, considered within context of living with a rare cancer.

### Implications for future research and daily practice

Future studies aiming to reduce unmet information needs and/or improve satisfaction among rare cancer survivors should involve repetitive measures of individual intervention components. This would enable greater understanding of the impact of each component over time and help elucidate possible influencers for outcome success.

Given the limited number of interventions targeting rare cancers that were delivered in an online, patient-led format, there is a need to develop and evaluate new, original interventions for this patient group. Future interventions should carefully incorporate and adapt the effective intervention components tested in common cancer patients, while also addressing the unique needs and challenges faced by rare cancer survivors. In particular, the benefit of, and preferences for, hard-copy, written material for the rare cancer survivor should be investigated. Moreover, more attention should be given to implementing interventions for rare cancers during the diagnosis and treatment phases of the cancer pathway, considering the limited evidence, yet challenges faced [4], in these timepoints.

Finally, health professionals involved in the management of rare cancers should be aware of the diverse and unique information needs of this population. Specifically, professionals should proactively seek their patients’ unmet information needs to ensure timely and relevant information provision as well as cultivate a sense of trust.

## Conclusion

Findings from this review provide key insights into current gaps in meeting the information needs and/or satisfaction with provided information for rare cancer survivors. Although important aspects of information delivery were identified, including how information should be provided and from whom, specific guidance is unclear for how this evidence, obtained primarily from common cancer survivors, should be used to inform interventions for rare cancer survivors. Future research should test these findings in a rare cancer context, because rare cancer survivors’ information needs, and satisfaction with information, appear to be compromised by the rarity of their condition and their challenging treatment trajectory. Ultimately, understanding and addressing information needs are crucial to enhancing overall well-being for all cancer survivors, including for those diagnosed with a rare cancer.

## Supplementary Information

Additional file1.

## Data Availability

All data generated or analysed during the current study have been cited in this published article. Search strategies for each database are provided in the Supplementary Information (Tables S1-S5).

## Abbreviation

RCT: Randomised controlled trial
