# Supplementary material for: Addressing cancer survivors’ information needs and satisfaction: a systematic review of potential intervention components for survivors with a rare cancer type
Source: Orphanet J Rare Dis. 2024 Oct 18;19:387. doi: 10.1186/s13023-024-03403-7 (PMC11488126; doi:10.1186/s13023-024-03403-7)
Supplement: Supplementary file 1 — Additional file1. [file 13023_2024_3403_MOESM1_ESM.docx]

**Farrugia et al. A systematic review of interventions addressing information needs among cancer survivors: can findings be translated to survivors with a rare cancer?**

Supplementary Information (Tables S1-S5) – Search strategy per database

Table S1 PubMed search strategy

| # | Search | Results |
| --- | --- | --- |
| 1 | Neoplasms[MeSH] | 2,894,460 |
| 2 | Cancer*[tiab] OR tumor*[tiab] OR tumour*[tiab] OR neoplasm*[tiab] OR neoplasia*[tiab] OR malignan*[tiab] OR benign[tiab] | 2,549,240 |
| 3 | 1 OR 2 | 3,390,797 |
| 4 | Intervention*[tiab] OR program*[tiab] | 938,003 |
| 5 | "information need"[tiab:~5] | 5,885 |
| 6 | "information needs"[tiab:~5] | 5,029 |
| 7 | “information provision”[tiab:~5] | 3,307 |
| 8 | 5 OR 6 OR 7 | 13,200 |
| 9 | "supportive care"[tiab] | 9,979 |
| 10 | “supportive need”[tiab:~5] OR “supportive needs”[tiab:~5] | 1,809 |
| 11 | 9 OR 10 | 10,790 |
| 12 | 8 OR 11 | 23,637 |
| 13 | 3 AND 4 AND 12 | 2,331 |
| 14 | 13 AND (8 AND “education*[tiab]”) | 2,873 |

Table S2 CINAHL search strategy

| # | Search | Results |
| --- | --- | --- |
| 1 | MH neoplasms | 91,280 |
| 2 | TI (cancer* OR tumor* OR tumour* OR neoplasm* OR neoplasia* OR malignan* OR benign)  OR AB (cancer* OR tumor* OR tumour* OR neoplasm* OR neoplasia* OR malignan* OR benign) | 654,976 |
| 3 | 1 OR 2 | 675,208 |
| 4 | TI intervention* OR AB intervention* | 546,972 |
| 5 | TI program* OR AB program* | 432,975 |
| 6 | TI education OR AB education | 392,759 |
| 7 | TI "information* need*" OR AB "information* need*" | 5,019 |
| 8 | TI information* N3 need* OR AB information* N3 need* | 15,282 |
| 9 | TI information N3 provision OR AB information N3 provision | 3,567 |
| 10 | TI “supportive care” OR AB “supportive care” | 7,416 |
| 11 | TI supportive N3 need* OR AB supportive N3 need* | 1,792 |
| 12 | 4 OR 5 OR 6 | 1,133,109 |
| 13 | 7 OR 8 OR 9 | 18,397 |
| 14 | 10 OR 11 | 8,282 |
| 15 | 3 AND 12 AND 13 AND 14 | 103 |
| 16 | Apply: 2011 – 2023, English language | 89 |

Table S3 PsycINFO search strategy

| # | Search | Results |
| --- | --- | --- |
| 1 | neoplasm/ | 59,594 |
| 2 | (cancer* OR tumor* OR tumour* OR neoplasm* OR neoplasia* OR malignan* OR benign).ti,ab | 93,869 |
| 3 | 1 OR 2 | 98,313 |
| 4 | intervention*.ti,ab | 456,221 |
| 5 | program*.ti,ab | 440,344 |
| 6 | education.ti,ab | 384,385 |
| 7 | information* need*.ti,ab | 3,619 |
| 8 | supportive care.ti,ab | 1,623 |
| 9 | 4 OR 5 OR 6 | 1,053,728 |
| 10 | 7 OR 8 | 5,191 |
| 11 | 3 AND 9 AND 10 | 628 |
| 12 | Apply: 2011 – 2023, English language, Humans | 427 |

Table S4 Embase search strategy

| # | Search | Results |
| --- | --- | --- |
| 1 | neoplasm/ | 511,869 |
| 2 | (cancer* OR tumor* OR tumour* OR neoplasm* OR neoplasia* OR malignan* OR benign).ti,ab | 5,335,779 |
| 3 | 1 OR 2 | 5,409,470 |
| 4 | intervention*.ti,ab | 216,990 |
| 5 | program*.ti,ab | 298,596 |
| 6 | education.ti,ab | 166,167 |
| 7 | information* need*.ti,ab | 12,262 |
| 8 | supportive care.ti,ab | 37,043 |
| 9 | 4 OR 5 OR 6 | 700,341 |
| 10 | 7 OR 8 | 49,022 |
| 11 | 3 AND 9 AND 10 | 1,028 |
| 12 | Apply: 2011 – 2023, English language, Humans | 831 |

Table S5 Cochrane Library search strategy

| # | Search | Results |
| --- | --- | --- |
| 1 | MeSH descriptor: [Neoplasm] explode all trees | 105,969 |
| 2 | (cancer* OR tumor* OR tumour* OR neoplasm* OR neoplasia* OR malignan* OR benign):ti,ab | 221,927 |
| 3 | 1 OR 2 | 248,942 |
| 4 | intervention*:ti,ab | 516,228 |
| 5 | program*:ti,ab | 143,036 |
| 6 | education:ti,ab | 59,246 |
| 7 | (information* need*):ti,ab | 20,383 |
| 8 | (supportive care):ti,ab | 6,416 |
| 9 | 4 OR 5 OR 6 | 590,592 |
| 10 | 7 OR 8 | 26,326 |
| 11 | 3 AND 9 AND 10 | 3,328 |
| 12 | Apply: 2011 – 2023, English language | 2,792 |
